# Supplementary material for: Kernel-based testing for single-cell differential analysis
Source: Genome Biol. 2024 May 3;25:114. doi: 10.1186/s13059-024-03255-1 (PMC11069218; doi:10.1186/s13059-024-03255-1)
Supplement: Supplementary file 1 — Additional file 1: Supplementary Material. [file 13059_2024_3255_MOESM1_ESM.pdf]

Kernel-Based Testing for Single-Cell Differential Analysis

A. Ozier-Lafontaine[*,1], C. Fourneaux[2], G. Durif[2], Polina Artenseva[1], C. Vallot[3,4], O. Gandrillon[2], S. Gonin-Giraud[2], B. Michel[†*,1], and F. Picard[*†,2]

[1]Nantes Université, Centrale Nantes, Laboratoire de Mathématiques Jean Leray, CNRS UMR 6629, F-44000, Nantes, France
[2]Laboratory of Biology and Modelling of the Cell, Université de Lyon, Ecole Normale Supérieure de Lyon, CNRS, UMR5239, Université Claude Bernard Lyon 1, Lyon, France
[3]CNRS UMR3244, Institut Curie, PSL University, Paris, France.
[4]Translational Research Department, Institut Curie, PSL University, Paris, France.

April 5, 2024

S.1 Supplementary Material

S.1 Full derivation of the Maximum Mean Discrepancy

We provide the full derivation of the Maximum Mean Discrepancy to explain how expected intra and inter-condition distances are involved in the definition of the statistic.

$$
\begin{aligned}
\mathrm{MMD}^2(\mu_1,\mu_2) &= \|\mu_1-\mu_2\|^2_{\mathcal{H}} \\
&= \langle\mu_1,\mu_1\rangle + \langle\mu_2,\mu_2\rangle - 2\langle\mu_1,\mu_2\rangle \\
&= \langle\mathbb{E}_{Y_1\sim\mathbb{P}_1}\big(\phi(Y_1)\big), \mathbb{E}_{Y_1'\sim\mathbb{P}_1}\big(\phi(Y_1')\big)\rangle + \langle\mathbb{E}_{Y_2\sim\mathbb{P}_2}\big(\phi(Y_2)\big), \mathbb{E}_{Y_2'\sim\mathbb{P}_2}\big(\phi(Y_2')\big)\rangle \\
&- 2\langle\mathbb{E}_{Y_1\sim\mathbb{P}_1}\big(\phi(Y_1)\big), \mathbb{E}_{Y_2\sim\mathbb{P}_2}\big(\phi(Y_2)\big)\rangle \\
&= \mathbb{E}_{Y_1\sim\mathbb{P}_1}\mathbb{E}_{Y_1'\sim\mathbb{P}_1}\langle\phi(Y_1),\phi(Y_1')\rangle + \mathbb{E}_{Y_2\sim\mathbb{P}_2}\mathbb{E}_{Y_2'\sim\mathbb{P}_2}\langle\phi(Y_2),\phi(Y_2')\rangle \\
&- 2\mathbb{E}_{Y_1\sim\mathbb{P}_1}\mathbb{E}_{Y_2\sim\mathbb{P}_2}\langle\phi(Y_1),\phi(Y_2)\rangle \\
&= \mathbb{E}_{Y_1\sim\mathbb{P}_1,Y_1'\sim\mathbb{P}_1}\left[k(Y_1,Y_1')\right] + \mathbb{E}_{Y_2\sim\mathbb{P}_2,Y_2'\sim\mathbb{P}_2}\left[k(Y_2,Y_2')\right] \\
&- 2\times\mathbb{E}_{Y_1\sim\mathbb{P}_1,Y_2\sim\mathbb{P}_2}\left[k(Y_1,Y_2)\right].
\end{aligned}
$$

S.2 Generalization to the multiple-conditions comparisons

Our approach can be generalized to the comparison of I conditions. Consider $I \geq 2$ groups of $n_1,\ldots,n_I$ observations (with $\sum_{i=1}^I n_i = n$) sucht that :

$$Y_{i,j} \sim \mathbb{P}_i, \quad i=1,\ldots,I \quad j=1,\ldots n_i.$$

For $i\in\{1,\ldots,I\}$, we denote by μ_i the kernel mean embedding of distribution $\mathbb{P}_i$ and $\mu=\sum_{i=1}^I n_i/n\mu_i$ is the kernel mean embedding of the distribution associated with the complete data. We can define the within-group covariance operator Σ_W and the between-group covariance operator Σ_B such that:

$$
\begin{aligned}
\Sigma_W &= \sum_{i=1}^I \frac{n_i}{n}\Sigma_i \\
\Sigma_B &= \sum_{i=1}^I \frac{n_i}{n^2}(\mu_i-\mu)^{\otimes 2}.
\end{aligned}
$$

Then, the test statistic has the same expression as the two-sample test statistic and asymptotically follows a Chi-square distribution with $(I-1)\times T$ degrees of freedom ([?], Chap 3). This discriminant approach

*To whom correspondence should be addressed: anthony.ozier-lafontaine@ec-nantes.fr, and also Bertrand.Michel@ec-nantes.fr, franck.picard@ens-lyon.fr
†joint last authors

with I conditions has $(I-1)$ discriminant directions defined as the $(I-1)$ first eigen-directions of the operator $(\Sigma_{W,T}^{-1}\Sigma_B)$. Note that when $I > 2$, the discriminant directions cannot be written explicitly with respect to the kernel mean embeddings, as in the two-sample case, which implies that the test statistic cannot be rewritten as a Mahalanobis distance.

S.3 Tuning the truncation hyperparameter

We use the simulation data to calibrate the hyperparameter of our method, i.e. the number T of principal directions of the within-covariance operator to retain to regularize the kernel-based Mahalanobis distance. The theoretical calibration of this hyperparameter still requires heavy mathematical developments, as shown by recent work [?]. However, these simulations provide a simple rule of thumb to choose it. Indeed, since T can be interpreted as the quantity of within-variance information used to describe the residual expression, increasing T will increase power in the detection of complex alternatives, at the price of increased type-I errors. In the simulations, Type-I errors of the kernel test remains at the nominal level $\alpha = 5\%$ until $T \leq 6$. with maximal power for $T = 4$ (Fig S.1). Interestingly, the test was completely unable to detect the DB alternative when $T = 1$. These results confirm that the truncation hyperparameter should be chosen as a trade-off between maximizing testing power while keeping the type-I errors controlled at the nominal level to ensure calibration. This motivates the choice of $T = 4$ for the univariate DE analyses in the simulations and the sc-RNASeq application. $T = 5$ was chosen for the sc-chIPSeq example.

For multivariate analyses, we assumed that the meaningful information was contained in more than four principal directions of the within-covariance operator and chose to take a larger truncation parameter in order to take into account more of the multivariate information available. We then chose the truncation parameter $T = 10$ for the sc-RNASeq example and $T = 5$ for the sc-chIPSeq data, that maximized the discriminant ratio while being not too large to still ensure the calibration.

S.4 Kernel trick for the effective computation of the test statistic

In this section, we describe how to compute the test statistic $\widehat{D}_T^2(\widehat{\mu}_1, \widehat{\mu}_2)$ and the vector of projections of the embeddings onto the discriminant axis V, with $i \in \{1,2\}$, $j \in \{1,\dots,n_i\}$, and $V = (\langle h_T^\star, \phi(Y_{i,j})\rangle_{\mathcal{H}})_{i,j}$ for $T \in \{1,\dots,n\}$. This computation relies on the kernel trick that consists in expressing every quantity of interest with respect to the gram matrix K containing every pair-wise evaluation of the kernel function, such that for $i, i' \in \{1,2\}$, $K = (K_{i,i'})_{i,i'}$, where for $j \in \{1,\dots,n_i\}$, $j' \in \{1,\dots,n_{i'}\}$, $K_{i,i'} = (k(Y_{i,j}, Y_{i',j'}))_{j,j'}$. The computation has two steps. First, we determine a matrix K_W that has the same eigenvalues as the operator $\widehat{\Sigma}_W$, then we compute the quantities of interest with respect to K, the T first eigenvalues $(\widehat{\lambda}_t)$, $t \in \{1,\dots,T\}$ and the associated unit eigenvectors $(u_t)_t$ of K_W. Let's denote by I_n the identity matrix of size n, J_n the matrix of size n full of 1, and $\mathbb{1}_n$ the vector of size n full of 1. Then for $i \in \{1,2\}$, let $P_i = I_{n_i} - n_i^{-1}J_{n_i}$, $P = \mathrm{diag}(P_1, P_2)$ and $\omega = (n_1^{-1}\mathbb{1}_{n_1}, -n_2^{-1}\mathbb{1}_{n_2})' \in \mathbb{R}^n$. We can show that the matrix K_W is equal to $K_W = n^{-1}PKP$. Then we have :

$$\widehat{D}_T^2(\widehat{\mu}_1, \widehat{\mu}_2) = \frac{n_1 n_2}{n^2}\sum_{t=1}^{T} \widehat{\lambda}_t^{-2}(u_t' PK\omega)^2, \quad \text{and} \quad V = \frac{n_1 n_2}{n^2}\sum_{t=1}^{T} \widehat{\lambda}_t^{-2}(u_t' PK\omega)KPu_t.$$

To correct for a batch effect, consider the case where the design shows B batches such that for $i \in \{1,2\}$ and $b \in \{1,\dots,B\}$, we have $n_{i,b}$ observations of condition i in batch b and $n_b = n_{1,b} + n_{2,b}$, then, for $i\{1,2\}$, we denote $Y_i = (Y_{i,1,1},\dots,Y_{i,1,n_{1,1}},\dots,Y_{i,B,1},\dots,Y_{i,B,n_{i,B}})$. To correct the batch effect in the feature space we considering the embedding $\widetilde{\phi}(Y_{i,b,j}) = \phi(Y_{i,b,j}) - n_b^{-1}\sum_{i'=1}^{2}\sum_{j'=1}^{n_{i',b}}\phi(Y_{i',b,j'})$ for $i \in \{1,2\}, b \in \{1,\dots,B\}$. In practice, this is done by replacing the Gram matrix K by the matrix $P_B K P_B$, where $P_B = (Q_{(i,b),(i',b')})_{i,i'\in\{1,2\},b,b'\in\{1,\dots,B\}}$, with

$$Q_{(i,b),(i',b')} \quad = \quad \begin{cases} I_{n_{i,b}} - n_b^{-1}J_{n_{i,b}} & \text{if } (i,b) = (i',b') \\ -n_b^{-1}J_{n_{i,b},n_{i',b}} & \text{if } i \neq i' \text{and } b = b' \\ 0 & \text{otherwise.} \end{cases}$$

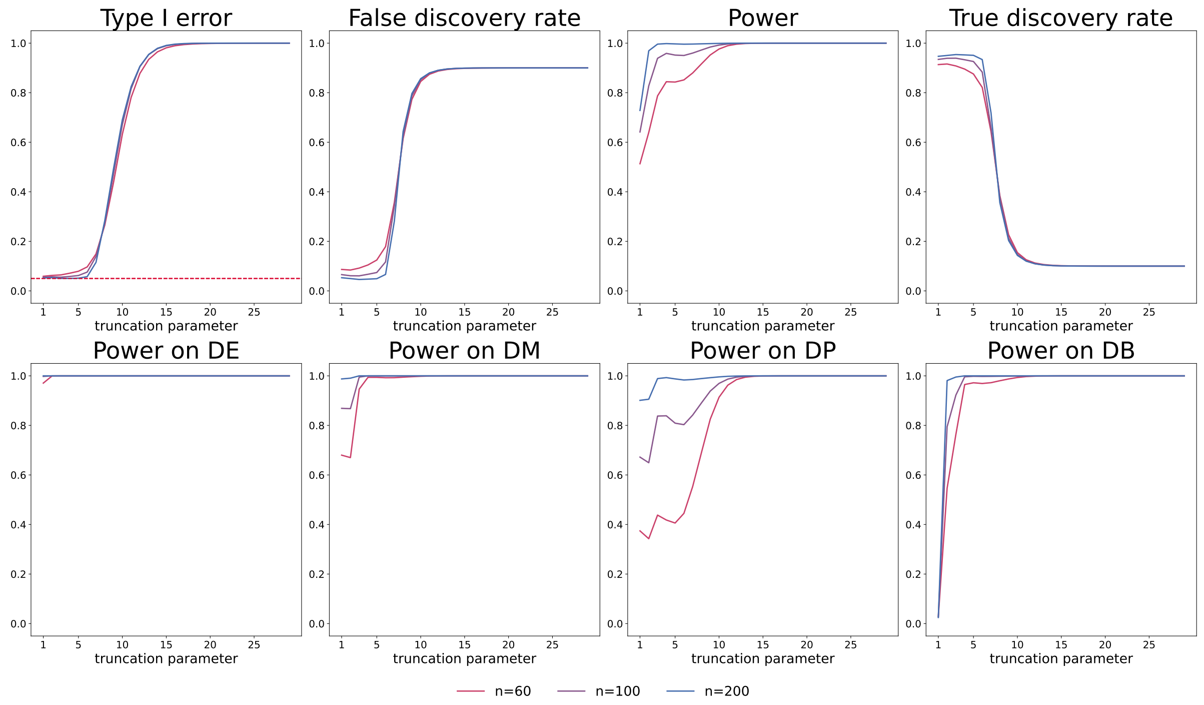

Figure S.1: Calibration of the truncation with respect to type-I errors and power. Top: Type-I errors are computed on raw p-values under H_0. False discovery Rate computed on Benjamini-Hochberg adjusted p-values. Power computed on raw p-values under H_1. True Discovery Rate computed on Benjamini-Hochberg adjusted p-values. Simulated data consists of 10000 genes (1000 DE, 9000 non-DE). Alternatives are simulated using DE : classical difference in expression (250 genes), DM : difference in modalities (250 genes), DP : difference in proportions (250 genes), DB : difference in both modalities and proportions with equal means (250 genes). Error rates are computed over 500 replicates.

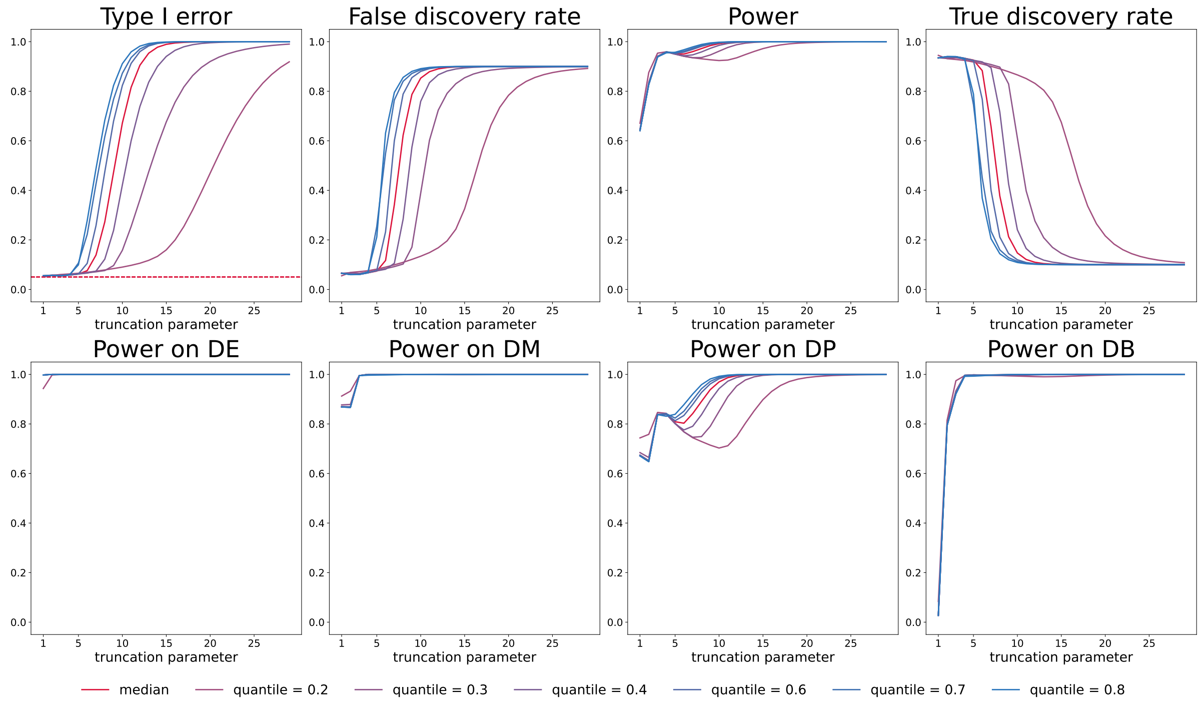

Figure S.2: Impact of the kernel's bandwidth on error-rates and power. Top: Type-I errors are computed on raw p-values under H_0. False discovery Rate computed on Benjamini-Hochberg adjusted p-values. Power computed on raw p-values under H_1. True Discovery Rate computed on Benjamini-Hochberg adjusted p-values. Simulated data consists of 10000 genes (1000 DE, 9000 non-DE). Alternatives are simulated using DE : classical difference in expression (250 genes), DM : difference in modalities (250 genes), DP : difference in proportions (250 genes), DB : difference in both modalities and proportions with equal means (250 genes). Error rates are computed over 500 replicates. The bandwidth is computed as the quantiles of the pairwise distances (as explained in Section **??** for the median).

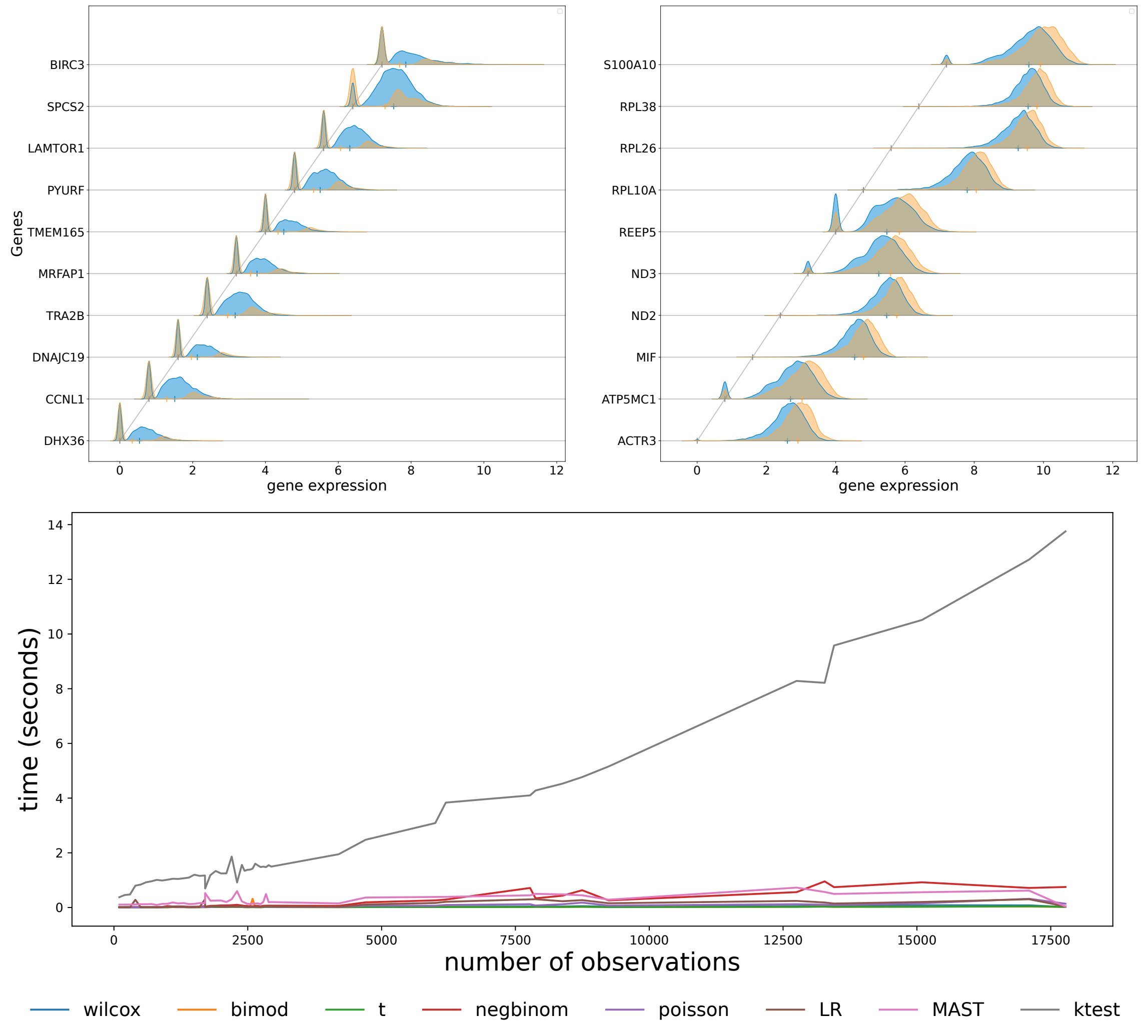

Figure S.3: Top: Expression densities of the two compared conditions for genes considered as DE by `ktest`-ZI-kernel and the other single-cell DE methods and considered as non-DE by pseudo-bulk methods. Left: stimulated memory Th0 cells (blue, 4766 cells) vs control memory Th0 cells (orange, 3110 cells) from [?]. Right : pig cells stimulated with lipopolysaccharide (blue, 6605 cells) vs control pig cells (orange, 6148 cells) from [?]. Bottom: average computing time (in seconds) of different DEA methods to analyse one gene, according to the number of cells in the sample.

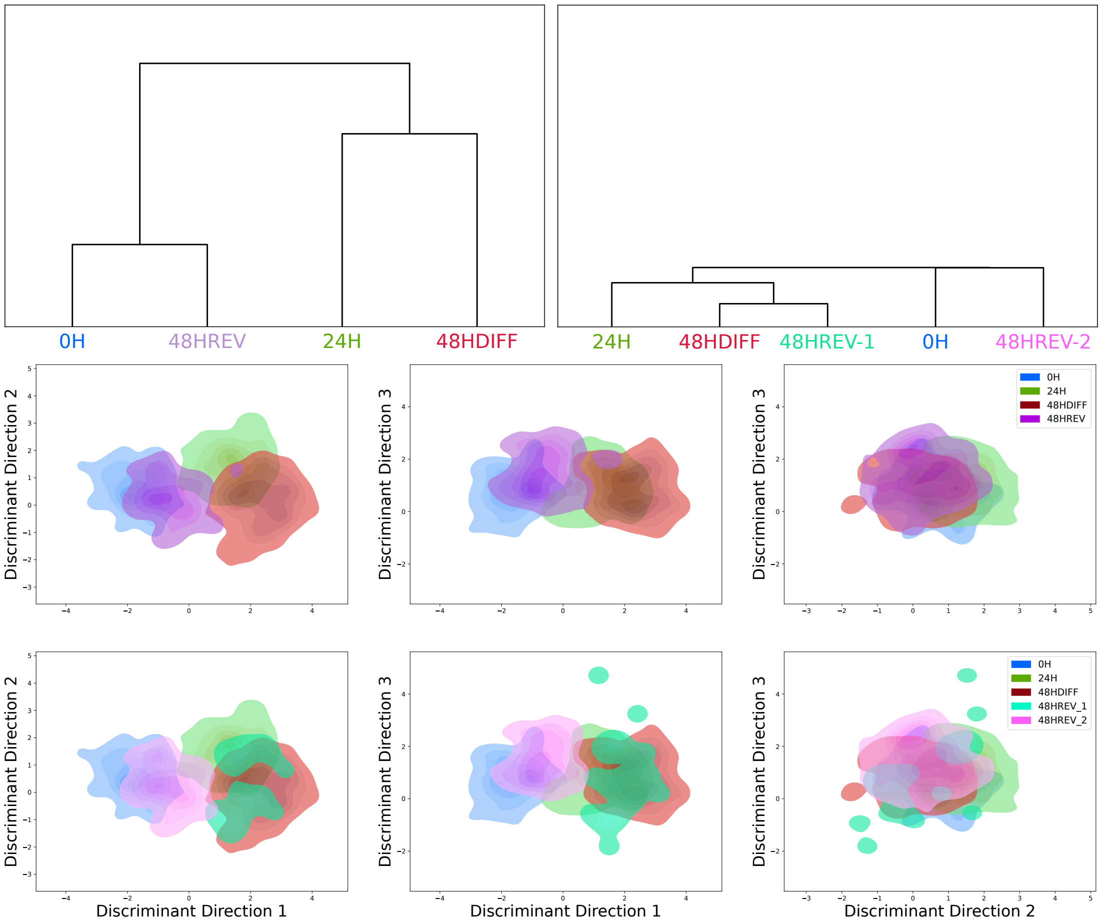

Figure S.4: Top : Trees from pairwise distances using our test statistic between conditions before (left) and after (right) splitting condition 48HREV into populations 48HREV-1 and 48HREV-2. Bottom : Cell densities of compared conditions projected on the 3 discriminant axes in the 4-group global comparison from RTqPCR-Seq data. The multivariate differential expression analysis was performed with $T = 10$.

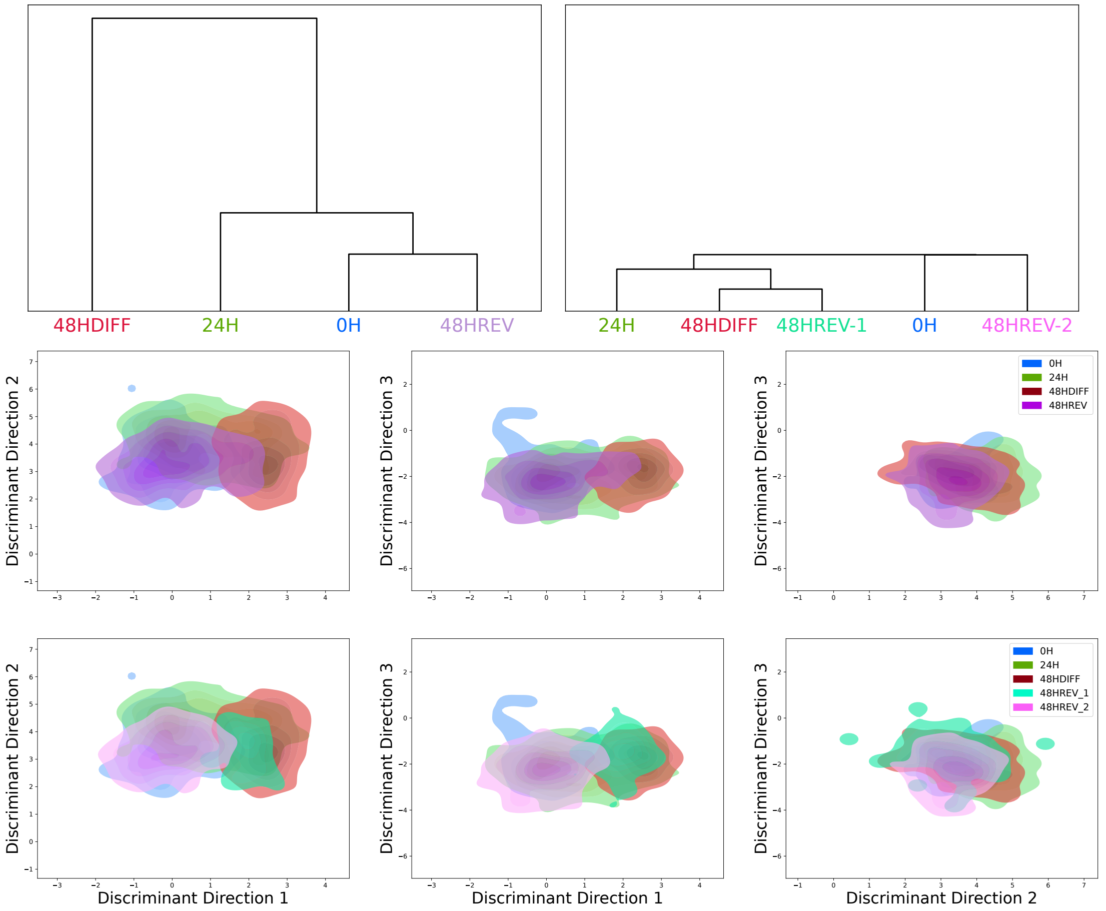

Figure S.5: Top : Trees from pairwise distances using our test statistic between conditions before (left) and after (right) splitting condition 48HREV into populations 48HREV-1 and 48HREV-2. Bottom : Cell densities of compared conditions projected on the 3 discriminant axes in the 4-group global comparison from scRNA-Seq data. The multivariate differential expression analysis was performed with $T = 10$.

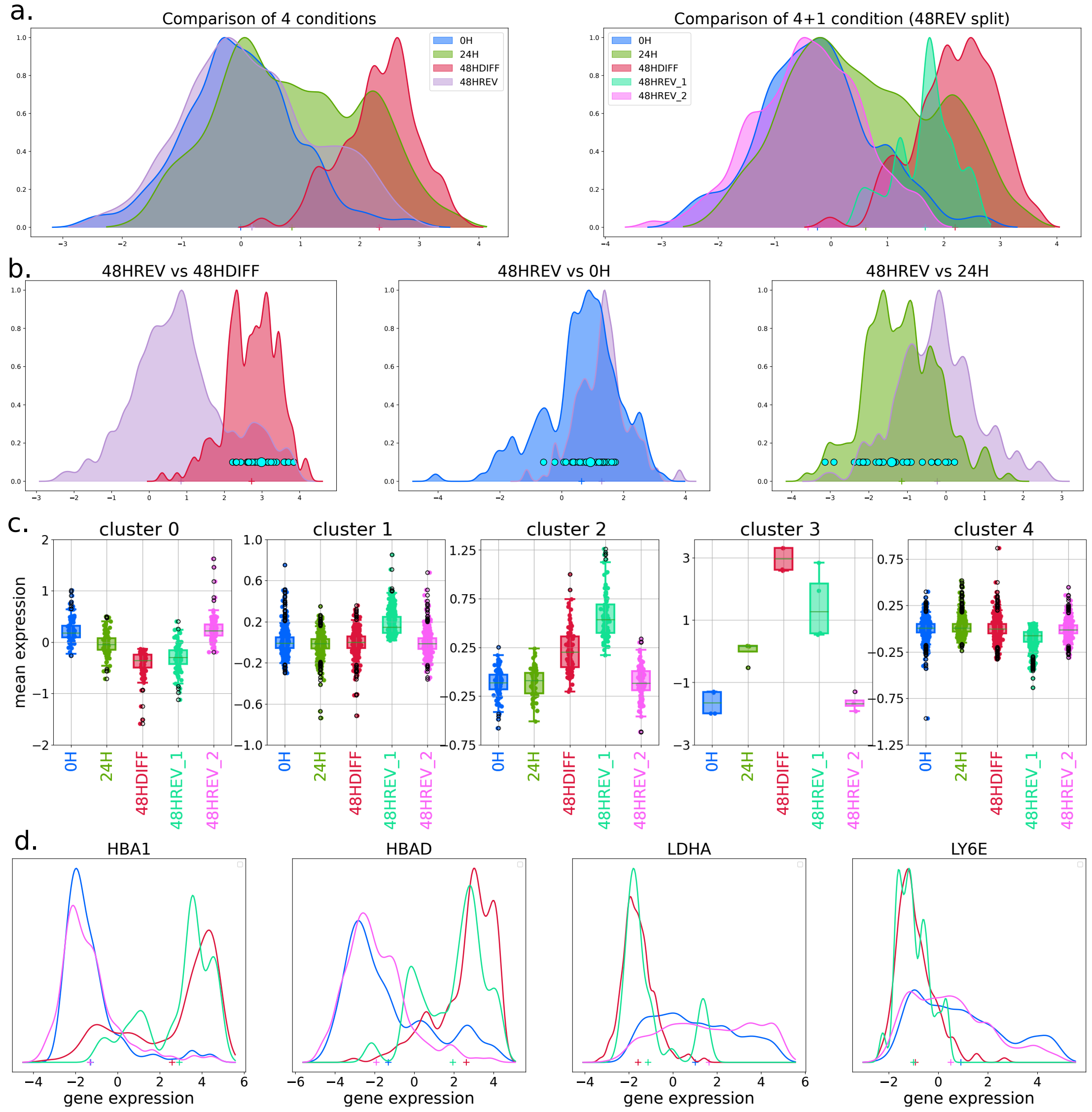

Figure S.6: a : Cell densities of compared conditions projected on the discriminant axis between conditions 48HREV and 48HDIFF (left), 48HREV and 0H (middle) and 48HREV and 24H (right) with highlighted population 48HREV-1. c: Boxplots of the variation of the gene expression along the five populations 0H, 24H, 48HDIFF, 48HREV-1 and 48HREV-2 for the five identified genes clusters.
d : Examples of gene expression distributions in populations 48HREV-1 (turquoise) and 48HREV-2 (pink) compared to populations 0H (blue) and 48HDIFF (red). a,b,c and d are obtained from scRNA-Seq data. The multivariate differential expression analysis was performed with $T = 10$.

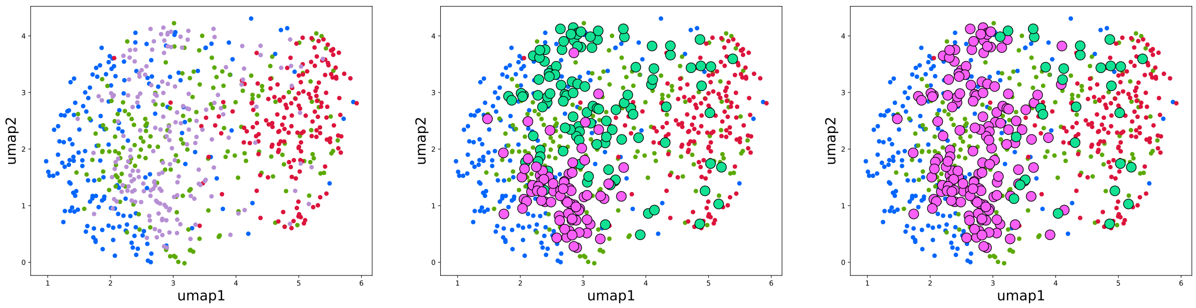

Figure S.7: Left: Umap representation of the four conditions from scRNA-Seq data (0H (blue), 24H (green) 48HDIFF (red) and 48HREV (purple)). Middle : highlight of the 2 groups of 48HREV identified through a k-means algorithm. Right : The two groups 48HREV-1 (turquoise) and 48HREV-2 (pink) identified on the discriminant axis associated to the truncation parameter $T = 10$.
