# Supplementary material for: Kernel-based testing for single-cell differential analysis
Source: Genome Biol. 2024 May 3;25:114. doi: 10.1186/s13059-024-03255-1 (PMC11069218; doi:10.1186/s13059-024-03255-1)
Supplement: Supplementary file 3 — Additional file 3: Review history. [file 13059_2024_3255_MOESM3_ESM.docx]

**1^st^ round**

**Reviewer 1**

The paper "Kernel-based testing for single-cell differential analysis" suggests a method for univariate and multivariate differential expression analysis based on kernel LDA (linear discriminant analysis). The authors argue that (1) in simulations, kPCA-based testing can have higher power than existing competitor methods; (2) in real data, visualisation aspect of LDA allows to uncover a "spectrum" of cells changing between two conditions, or a heterogeneity within one of the predefined groups.

Disclaimer: I am not a specialist in differential expression testing and am not entirely familiar with recent literature. Therefore I am unable to judge to what extent the authors make all the relevant comparisons, and to what extent their method is novel.

That said, I think the paper is interesting and quite clearly written. I mostly have only minor comments. In terms of improving state of the art, the suggested method performs better than competitors (higher power) in some simulations but not in others. So here the results are somewhat mixed. But the visualization aspect (projection on the discriminat axis) I found interesting.


ISSUES

* lines 116-126: This selling point is overstating the results, in my opinion. I assume it refers to the zi-Gaussian kernel, but the Results show that it does NOT perform any better than the standard Gaussian kernel. So I find it overselling to say that the suggested method takes advantage of probablistic models of scRNA-seq data.

* lines 323-325: I don't understand how this is the same equation as the previous one in line 320. Why would |mu1 - mu2|^2 involve terms like within-condition similarity?

* Section 2.1 introduces Mahalanobis distance, and then section 2.2 presents LDA, arriving to the formula, and it seems unnecessary repetitive. Maybe consider merging these two sections together, or alternatively make it clearer why they are separate. The presentation logic can be improved here.

* line 380: I would suggest to describe here in more details the effect of truncation of the SVD of Sigma_W. First, how do you invert Sigma_W which is not full rank? Please spell it out. Second, AFAIK this should have an effect similar to regularization; if so, please write explicitly, that this is a way to regularize the LDA, in particular in d>>n sitation (which is always the case for kernel LDA, as d = infty). Third, please mention the T choice in the main text. As becomes clear only in the Methods, you use T=4 for univariate and T=10 for multivariate tests.

* line 521: what is the "median heuristic"? Please describe briefly.

* line 522: "zero-inflation is another important characteristic of scRNA-Seq data" -- this sentence lacks evidence or citations. I would suggest to add some citations here. But note that for UMI-based scRNA-seq data this is not correct: see https://www.nature.com/articles/s41587-019-0379-5, https://www.nature.com/articles/s41587-020-00810-6, and many other recent works. Zero-inflation (compared to negative binomial) only concers non-UMI data (like e.g. Smart-Seq). Please make it clear, what kind of scRNA-seq data this sentence is referring to, and what kind of data you will later test it on (UMI or read count).

* line 526: "zero-inflated Gaussian": nobody models scRNA-seq data as zero-inflated Gaussian, and in fact when you do the simulations you use zero-inflated negative binomial. Why assuming zero-inflated Gaussian in section 2.3 is a reasonable choice?

* line 553: "Korthauer's alternatives": what is this? Please describe here in detail what this term means.

* section 2.4 does not describe or even list the methods that you compare your method with. I suggest to list and cite all methods here in main text, and to give a brief one-sentence description of each of them! Are all of these methods "multivariate"?

* section 2.5 I found strangely inconclusive. The authors argue that it is not correct to use bulk sequencing as ground truth, so in the end it is impossible to say which method performs better. Also, if conclusions of Ref [53] are not to be trusted, then why is "over-detecting highly-expressed genes" (line 619) a bad thing (bias)?

* There is some inconsistency between conclusiond drawn from Fig 3 top and bottom. Fig 3top is interpreted as kernel testing being close to single-cell methods. But then in Fig 3bottom we see that kerel testing behaves differently from single-cell methods and in fact seems more similar to bulk/pseudobulk. Why is that?

* Does Fig 3 use a different set of comparison methods compared to Fig 2? Why? If yes, please list and describe all new methods here.

* Figure 2: what is your method? I assume it is "kfda", but this name is not mentioned in the main text, so that can be a bit confusing.

* Have you experimented with LDA with scalar product kernel (i.e. non-kernel, standard LDA)? Does it perform worse than Gaussian? That would be nice to see. Otherwise one can wonder, why you are not using standard LDA.

* Multiclass LDA seems like an obvious extension, and could be used for the dataset from Fig 4. Then one could visualize the data by projecting all the cells to 2D. That would be nice.

**Reviewer 2**

Ozier-Lafontaine et al provide a differential analysis framework that can be used for multiple types of single-cell genomic data (gene expression, epigenetic, DNA). The authors seek to complement existing methods for differential analysis, and specifically aim to overcome limitations of parametric, gene-wise two-sample statistical tests, which are not always suited to the variety of distributions present in single-cell data. To account for the non-linear relationships among genes that can arise in response to perturbations due to genetic interactions and the hierarchical structure of gene regulatory networks, the authors have used kernel-based embedding in feature-space. Crucially, these embeddings can be described in terms of means and variances, enabling statistical testing to identify of global differences between treatments. Using a test statistic derived from distances between the embeddings for different groups of cells, the method allows hypothesis testing beyond gene-by-gene. The approach is benchmarked against many conventional DE testing approaches, and performs well, with less bias toward highly expressed genes and with robustness to zero-inflation. Applying their method to existing datasets, the authors find previously uncharacterized cell stats that can be associated with gene expression differences in the original feature space. With the Python and R package, the testing framework presented seems accessible and immediately useful. While I cannot comment on the precise implementation of the kernel-based approach from a software perspective, it appears to have a reasonable run-time. Overall, the authors provide a worthwhile, complementary approach to typical gene-by-gene testing in single-cell analysis while maintaining interpretability of the global differences detected.


Major Comments

While the authors make an earnest nod in the Discussion toward further progress in applying this method for batch correction, I was surprised there was no attempt to show this, given the immediately utility of such an approach. The same arguments proposed by the authors for non-linear relationships among genes between a treated and untreated sample would also apply for two datasets generated with slightly different techniques, or due to some other source of batch variation.

The kernel-based approach seems to perform exceptionally well in detecting cases of different modality and proportion but equal mean expression (DB). Could you add detail on the relative frequency of this case (in certain kinds of samples or responses in gene regulatory networks, etc), to highlight the "blindspots" that your method will help illuminate? Additionally, how frequently was the DB pattern detected in the real data used in this study?

It would be useful to have more detail for the compute times with this method. Perhaps associated with Figure 3, some kind of comparison could be made along with the other benchmarking results.

How are results affected by the bandwidth parameter of the kernel function? Should the user expect to adjust this or apply the same heuristic referenced in the manuscript?

Could you elaborate further on the future applications (and challenges) for multi-sample comparisons using this method in the Discussion?


Minor Comments

The color and quality of the lines in Figure 2 are very hard to read, both in the plots and in the legend.


"Thanks to a very fruitful collaboration/data sharing with colleagues having developed a competing method [14],"

This is a nice sentiment, but this strangely informal sentence sounds like it belongs in the acknowledgements. Could you instead highlight the existence of a similar method, and comment on any differences between the two before moving onto the zero-inflation results?

**Authors’ response**

Reviewer 1

The paper "Kernel-based testing for single-cell differential analysis" suggests a method for univariate and multivariate differential expression analysis based on kernel LDA (linear discriminant analysis). The authors argue that (1) in sim- ulations, kPCA-based testing can have higher power than existing competitor methods; (2) in real data, visualisation aspect of LDA allows to uncover a "spectrum" of cells changing between two conditions, or a heterogeneity within one of the predefined groups.

Disclaimer: I am not a specialist in differential expression testing and am not entirely familiar with recent literature.

Therefore I am unable to judge to what extent the authors make all the relevant comparisons, and to what extent their method is novel. That said, I think the paper is interesting and quite clearly written. I mostly have only minor comments. In terms of improving state of the art, the suggested method performs better than competitors (higher power) in some simulations but not in others. So here the results are somewhat mixed. But the visualization aspect (projection on the discriminant axis) I found interesting.

Answer: Thank you for giving us the opportunity to submit a revised version of our work. Your remarks and questions have truly helped us improve the quality of the manuscript, and have allowed us to broaden the scope of our method.

We hope you will find our answers convincing.

1. lines 116-126: This selling point is overstating the results, in my opinion. I assume it refers to the zi-Gaussian kernel, but the Results show that it does NOT perform any better than the standard Gaussian kernel. So I find it overselling to say that the suggested method takes advantage of probabilistic models of scRNA-seq data.

Answer: We agree with Referee 1, and have now tempered our sentence Page 2 line 55.

2. lines 323-325: I don’t understand how this is the same equation as the previous one in line 320. Why would ‖μ1 − μ2‖2 involve terms like within-condition similarity?

Answer: We now propose a full derivation of the MMD statistic. Details are provided in the Supplementary Material Page 16.

3. Section 2.1 introduces Mahalanobis distance, and then section 2.2 presents LDA, arriving to the formula, and it seems unnecessary repetitive. Maybe consider merging these two sections together, or alternatively make it clearer why they are separate. The presentation logic can be improved here.

Answer: We acknowledge the concerns raised by Referee 1 regarding our presentation. Our intention is to present our test statistic as a Mahalanobis distance, which could be easily interpreted in terms of the difference in mean embeddings normalized by some variance. Also, this first step allows us to introduce regularization. However, we believe that, in this initial step, the LDA framework is not essential for comprehending the test. This is why we choose the title "Testing with a Mahalanobis distance between gene-expression embeddings". Then, in a second part, we explain the connection with the LDA framework and the interpretation of the dis- criminant axis, that provides an additional visualization tool for our procedure, and allows its generalization to multiple comparisons. Hence, we would like to keep this two-step explanation because we think that the reader may not be familiar with the framework (and it provides two balanced subsections in the text).

4. line 380: I would suggest to describe here in more details the effect of truncation of the SVD of ΣW . First, how do you invert ΣW which is not full rank? Please spell it out. Second, AFAIK this should have an effect similar to regularization; if so, please write explicitly, that this is a way to regularize the LDA, in particular in d >> n situation (which is always the case for kernel LDA, as d = ∞). Third, please mention the T choice in the main text. As becomes clear only in the Methods, you use T = 4 for univariate and T = 10 for multivariate tests.

Answer: We now propose a reformulation of the regularization strategy Page 3 Line 150. The choice of T is now explicitly mentioned in all analysis and figures.

5. line 521: what is the "median heuristic"? Please describe briefly.

Answer: This is now detailed briefly in Section 2.3 Page 5 line 231.

6. line 522: "zero-inflation is another important characteristic of scRNA-Seq data" – this sentence lacks evidence or citations. I would suggest to add some citations here. But note that for UMI-based scRNA-seq data this is not correct: see https://www.nature.com/articles/s41587-019-0379-5, https://www.nature.com/articles/s41587-020- 00810-6, and many other recent works. Zero-inflation (compared to negative binomial) only concers non-UMI data (like e.g. Smart-Seq). Please make it clear, what kind of scRNA-seq data this sentence is referring to, and what kind of data you will later test it on (UMI or read count).

Answer: This was unclear indeed, and we totally agree. We added the reference and now present this ZI-Gauss kernel in a different way. This was a clumsiness of ours to introduce the ZI-Gauss distribution, whereas we were only interested in the potential excess of zeros in the data that could alter the calibration of the Gaussian kernel’s bandwidth. This has been reformulated Page 5 line 233, and Page 9 line 456.

7. line 526: "zero-inflated Gaussian": nobody models scRNA-seq data as zero-inflated Gaussian, and in fact when you do the simulations you use zero-inflated negative binomial. Why assuming zero-inflated Gaussian in section 2.3 is a reasonable choice?

Answer: Indeed. This has been re-written because we do not assume any distribution for the data. The proba- bility product kernel is just a framework to derive a new kernel as now explained Page 9 line 456.

8. line 553: "Korthauer’s alternatives": what is this? Please describe here in detail what this term means.

Answer: We reformulated this sentence Page 6 line 247.

9. section 2.4 does not describe or even list the methods that you compare your method with. I suggest to list and cite all methods here in main text, and to give a brief one-sentence description of each of them! Are all of these methods "multivariate"?

Answer: Methods are now shortly described in Supp. Tables S1 and S2 (Page 18). Also, our framework is the only one to be able to perform multivariate comparisons (as well as univariate tests), and that all methods comparisons are based on univariate tests.

10. section 2.5 I found strangely inconclusive. The authors argue that it is not correct to use bulk sequencing as ground truth, so in the end it is impossible to say which method performs better. Also, if conclusions of Ref [53] are not to be trusted, then why is "over-detecting highly-expressed genes" (line 619) a bad thing (bias)?

Answer: We perfectly agree with Reviewer 1 that taking the bulk results as a ground truth makes the comparison challenging. We reformulated the ’bias’ into a more moderate sentence (Page 6 lines 284).

11. There is some inconsistency between conclusion drawn from Fig 3 top and bottom. Fig 3 top is interpreted as kernel testing being close to single-cell methods. But then in Fig 3bottom we see that kernel testing behaves differently from single-cell methods and in fact seems more similar to bulk/pseudobulk. Why is that?

Answer: The top panel of Fig. 3 indicates indeed that, in terms of error rates, ktest lies between single-cell and pseudo-bulk methods. Fig. 3 bottom shows that among the top 500 DE genes detected by each method, a large proportion show higher expression and lower proportion of zeros for single-cell methods as compared with pseudo-bulk and ktest. We do not have a definitive answer to Referee 1’s question, but one explanation could be that pseudo bulk methods are more robust and more accurate to estimate the overdispersion of the data (reason for which they are used), and that the median heuristic we use to calibrate the width of the kernel in ktest, combined with non-linearity, ensures some robustness to this overdispersion.

12. Does Fig 3 use a different set of comparison methods compared to Fig 2? Why? If yes, please list and describe all new methods here.

Answer: Clarifications were needed indeed. Fig.2 corresponds to simulated data as proposed by Gauthier et al., and Fig. 3 to the analysis of scRNA-Seq data as provided by Squair et al. Since the two parts are independent and referring to different references, we did not cross the methods between sections. But we now provide supplementary Tables S1-S2 that describe briefly the methods compared on simulated data and on scRNA-Seq data (page 18).

13. Figure 2: what is your method? I assume it is "kfda", but this name is not mentioned in the main text, so that can be a bit confusing.

Answer: This has been changed.

14. Have you experimented with LDA with scalar product kernel (i.e. non-kernel, standard LDA)? Does it perform worse than Gaussian? That would be nice to see. Otherwise one can wonder, why you are not using standard LDA.

Answer: We now added the results of the linear kernel compared with other methods on simulated data (Fig. 1) and experimental data (Fig 2.). Fig 1 clearly shows that the power of the linear method is lower. Fig. 2 shows that the performance of the linear kernel is close to the t-test. This has been added in the main text page 5 line 243, page 6 line 284.

15. Multiclass LDA seems like an obvious extension, and could be used for the dataset from Fig 4. Then one could visualize the data by projecting all the cells to 2D. That would be nice.

Answer: Indeed, it was not difficult to generalize ktest to the multigroup approach for the reversion data. Methodological developments are provided in the Supp. Mat. (page 16 line 531), and mentioned in the main text (page 5 line 217). The new analysis is provided Page 7 line 321, along with Fig. 4 page 13, Fig S9 page 20, Fig S10 page 21. We now analyze the 4 conditions using the multigroup comparison first (Fig 4b, page 13) and then pursue with pair-wise comparisons (Fig. 4c, page 13). Thanks to the suggestion of Referee 2, we also provide a correction of the batch effect in the feature space, allowing our method to handle more general designs, as now mentioned in the introduction (Page 2 line 95).

Reviewer 2

Ozier-Lafontaine et al provide a differential analysis framework that can be used for multiple types of single-cell genomic data (gene expression, epigenetic, DNA). The authors seek to complement existing methods for differential analysis, and specifically aim to overcome limitations of parametric, gene-wise two-sample statistical tests, which are not always suited to the variety of distributions present in single-cell data. To account for the non-linear relationships among genes that can arise in response to perturbations due to genetic interactions and the hierarchical structure of gene regulatory networks, the authors have used kernel-based embedding in feature-space. Crucially, these embed- dings can be described in terms of means and variances, enabling statistical testing to identify of global differences between treatments. Using a test statistic derived from distances between the embeddings for different groups of cells, the method allows hypothesis testing beyond gene-by-gene. The approach is benchmarked against many conventional DE testing approaches, and performs well, with less bias toward highly expressed genes and with robustness to zero- inflation. Applying their method to existing datasets, the authors find previously uncharacterized cell stats that can be associated with gene expression differences in the original feature space. With the Python and R package, the testing framework presented seems accessible and immediately useful. While I cannot comment on the precise implementa- tion of the kernel-based approach from a software perspective, it appears to have a reasonable run-time. Overall, the authors provide a worthwhile, complementary approach to typical gene-by-gene testing in single-cell analysis while maintaining interpretability of the global differences detected.

Answer: Thank you for giving us the opportunity to submit a revised version of our work. Your remarks and questions have truly helped us improve the quality of the manuscript, and have allowed us to broaden the scope of our method.

We hope you will find our answers convincing.

Major comments

1. While the authors make an earnest nod in the Discussion toward further progress in applying this method for batch correction, I was surprised there was no attempt to show this, given the immediately utility of such an approach. The same arguments proposed by the authors for non-linear relationships among genes between a treated and untreated sample would also apply for two datasets generated with slightly different techniques, or due to some other source of batch variation.

Answer: It is indeed possible to correct for batch effects in a non-linear manner. We now propose to illus- trate this strategy on the reversion dataset, for which the design contains 4 batches. Methodological details are provided in Supp. Mat Page 17 line 577. The batch effect correction did not change the conclusions on the biological conditions comparisons in this example. Thanks to Referee’s 1 comment, we also added the multi- group comparisons Page 7 line 321, and mentioned in the introduction that our method can handle more general designs (Page 2 line 95).

2. The kernel-based approach seems to perform exceptionally well in detecting cases of different modality and proportion but equal mean expression (DB). Could you add detail on the relative frequency of this case (in certain kinds of samples or responses in gene regulatory networks, etc), to highlight the "blindspots" that your method will help illuminate? Additionally, how frequently was the DB pattern detected in the real data used in this study?

Answer: Your suggestion is very interesting, but since our method does not provide any typology of the rejected hypothesis (ie whereas the rejection is due to a DB-type of alternative for instance), it appears very difficult to assess the relative frequency of the DB scenario. However, what we can say is that the DB alternative typically corresponds to differential abundance scenarios ( ie only a difference in the relative number of cells ), which has been the subject to important methodological developments (see for instance: Differential abundance testing on single-cell data using k-nearest neighbor graphs, Dann et al, 2022).

3. It would be useful to have more detail for the compute times with this method. Perhaps associated with Figure 3, some kind of comparison could be made along with the other benchmarking results.

Answer: This figure has been added page 19 Figure S8, and discussed page 6 line 298. The figure shows the quadratic complexity of the kernel method, which remains scalable thanks to the Nystrom approximation.

4. How are results affected by the bandwidth parameter of the kernel function? Should the user expect to adjust this or apply the same heuristic referenced in the manuscript?

Answer: We added the performance of our method for varying bandwidth (Fig. S7 page 18) on simulated data, which shows that the median heuristic establishes a good trade-off between type-I/power. The new figure shows that there is a connection between the bandwidth of the kernel and the truncation parameter, as now mentioned page 6 line 264.

5. Could you elaborate further on the future applications (and challenges) for multi-sample comparisons using this method in the Discussion?

Answer: As we now propose the application for multiple group comparisons and a two-way design, we have simplified the conclusion of our manuscript. Although there are still some developments to be made in this direction, the core idea is to develop a linear model in the feature space. Thus, we need to assess the testability of general hypotheses in the Reproducing Kernel Hilbert Space and, for instance, investigate the homoscedasticity hypothesis in the feature space. We acknowledge that these matters are somewhat theoretical and may not be central to the current manuscript. Given the substantial material added to the document, we suggest simplifying the conclusion (page 9 line 426).

Minor comments

1. The color and quality of the lines in Figure 2 are very hard to read, both in the plots and in the legend.

Answer: This has been changed, we hope the figure is easier to read.

2. "Thanks to a very fruitful collaboration/data sharing with colleagues having developed a competing method [14]," This is a nice sentiment, but this strangely informal sentence sounds like it belongs in the acknowledge- ments. Could you instead highlight the existence of a similar method, and comment on any differences between the two before moving onto the zero-inflation results?

Answer: We agree with Referee 2 on the informal sentence, and kept it for the Acknowledgments. We now provide the list of methods compared in Tables S1 and S2 page 18.

**2^nd^ round**

**Reviewer 1**

Thanks for addressing the comments.

**Reviewer 2**

The authors have addressed all of my concerns and I appreciate the clarity of both the batch-correction analysis and new figures.
